# Cooperative Stimulation of Megakaryocytic Differentiation by Gfi1b Gene Targets Kindlin3 and Talin1

**DOI:** 10.1371/journal.pone.0164506

**Published:** 2016-10-21

**Authors:** Divya Singh, Ghanshyam Upadhyay, Ananya Sengupta, Mohammed A. Biplob, Shaleen Chakyayil, Tiji George, Shireen Saleque

**Affiliations:** Department of Biology, The City College of New York and The Graduate Center of The City University of New York, 160 Convent Avenue, New York, NY, 10031, United States of America; B.C. Cancer Agency, CANADA

## Abstract

Understanding the production and differentiation of megakaryocytes from progenitors is crucial for realizing the biology and functions of these vital cells. Previous gene ablation studies demonstrated the essential role of the transcriptional repressor Gfi1b (growth factor independence 1b) in the generation of both erythroid and megakaryocytic cells. However, our recent work has demonstrated the down-regulation of this factor during megakaryocytic differentiation. In this study we identify two new gene targets of Gfi1b, the cytoskeletal proteins Kindlin3 and Talin1, and demonstrate the inverse expression and functions of these cytoskeletal targets relative to Gfi1b, during megakaryocytic differentiation. Both *kindlin3* and *talin1* promoters exhibit dose dependent Gfi1b and LSD1 (lysine specific demethylase 1; a Gfi1b cofactor) enrichment in megakaryocytes and repression in non-hematopoietic cells. Accordingly the expression of these genes is elevated in *gfi1b* mutant and LSD1 inhibited hematopoietic cells, while during megakaryocytic differentiation, declining Gfi1b levels fostered the reciprocal upregulation of these cytoskeletal factors. Concordantly, manipulation of Kindlin3 and Talin1 expression demonstrated positive correlation with megakaryocytic differentiation with over-expression stimulating, and inhibition diminishing, this process. Co-operativity between these factors and integrins in promoting differentiation was further underscored by physical interactions between them and integrinβ3/CD61 and by stimulation of differentiation by the Talin1 head domain, which is necessary and sufficient for integrin activation. Therefore this study demonstrates the significance of Gfi1b regulated Kindlin3-Talin1 expression in driving megakaryocytic differentiation and highlights the contribution of cytoskeletal agents in the developmental progression of these platelet progenitors.

## Introduction

Growth factor independence 1b (Gfi1b) is a zinc finger transcriptional repressor essential for generation of the erythroid and megakaryocytic lineages during embryonic development [1] and in adults [2]. Accordingly, deletion of *gfi1b* either in the germ line or in adult hematopoietic stem cells (HSCs) swiftly leads to lethal anemia due to an arrest in erythroid development. Gfi1b has also been implicated in lymphoid development [3–5] and is moderately expressed in multiple non-hematopoietic tissues [6, 7]. However its specific contribution if any, to other developmental processes awaits the conditional deletion of the floxed gene [2] either in specific hematopoietic lineages or in non-hematopoietic cells. Dysregulation of Gfi1b, and/or its paralog Gfi1, have also been observed in various hematopoietic and non-hematopoietic malignancies [8–12] particularly in erythroleukemias and megakaryocytic leukemias [9]. Interestingly, a recent report causally linked a dominant negative nonsense mutation in Gfi1b with a type of “Gray platelet syndrome” characterized by dysmorphic megakaryocytes and abnormal and functionally impaired platelets [13].

Both Gfi1 and Gfi1b have been reported to bind the DNA motif TAAATCAC(A/T)GCA [6, 14] and mediate transcriptional repression of target loci by recruiting chromatin modifiers and co-repressors such as LSD1 (lysine specific demethylase1; Kdm1a), Rcors1-2 (REST corepressors1-2), HDAC1/2 (histone deacetylase1/2) and histone methyl transferases such as G9a and SUV39H1 [15–18]. To elucidate the mechanism of action of Gfi1b and its major cofactors, LSD1 and Rcor1/CoREST, in mediating erythro-megakaryocytic differentiation, chromatin immunoprecipitation screens (ChIP on chip) were previously performed for Gfi1b, LSD1 and Rcor1/CoREST in erythroid cells [15]. This triple ChIP approach lead to the identification of >600 putative common ChIP targets which were further sorted by their extent of derepression in LSD1 inhibited erythroid cells relative to controls [15]. The combination of these ChIP and microarray profiling screens uncovered some new and interesting gene targets including the oncogene and transcription factor *meis1* [19], the signaling molecule *rgs18* (regulator of G protein signaling 18) [20] and the cytoskeletal protein genes *kindlin3* and *talin1* (this report).

Kindlin3/URP2/FERMT3 is a one of three members of a family of cytoskeletal proteins that is primarily expressed in hematopoietic cells especially in platelets, megakaryocytes and erythroid cells [21]. Accordingly, deletion of the *kind3/Fermt3* gene produces perinatal lethality due to severe hemorraging [22] and anemia [23]. These phenotypes result from defects in platelet activation and erythrocyte maturation which in turn reflect either a failure of integrin activation in platelets [22] or aberrant membrane protein organization in erythrocytes [23], respectively. Talin1 is another cytoskeletal protein that exhibits more widespread tissue expression. Concordantly, the *talin1* germline deletion produces peri-implantation lethality due to major defects in cell migration during gastrulation [24]. In contrast, conditional deletion of *talin1* in hematopoietic cells mediated by Mx1-CRE mediated elimination of the floxed allele in HSCs exhibited defects in platelet activation and function without noticeably impacting other lineages or viability [25]. Both Kindlin3 and Talin1 are known to bind to the cytoplasmic tails of integrins particularly the β3 subunit of αIIbβ3 integrins, which are most abundantly expressed in platelets [26–28]. These interactions lead to conformational changes in the transmembrane (tilting) and extracellular (unfurling) domains respectively, leading to “inside out” activation of the integrin molecule thereby greatly increasing their affinity for ligands such as fibrinogen. This integrin activation and ligand binding then enables “outside-in” signaling that produces the requisite spreading and aggregation of platelets during thrombus formation [26, 28]. However, neither platelet counts nor megakaryocyte numbers were reported to be altered in *kindlin3* and *talin1* deficient animals, implying minimal or no functional impact of these proteins at earlier developmental stages [22, 23].

Following the identification of the *kindlin3* and *talin1* loci as potential Gfi1b/LSD1/Rcor targets we confirmed that Gfi1b is both recruited to, and represses, these promoters in a dose dependent manner. Accordingly, both targets were upregulated in *gfi1b-/-* and LSD1 inhibited hematopoietic cells and along with integrinβ3 exhibited an inverse expression pattern relative to Gfi1b in maturing megakaryocytes. Manipulation of Kindlin3 and Talin1 expression further demonstrated the stimulatory effect of these proteins on megakaryocytic differentiation and vice versa. These observations demonstrate that upregulation of the cytoskeletal proteins Kindlin3, Talin1 and integrins actively promote megakaryocytic differentiation likely by remodeling their cytoskeletons and priming them for platelet production. The results presented here therefore add these cytoskeletal proteins to the growing list of Gfi1b targets like Meis1 [19] and Rgs18 [20] whose reciprocal up-regulation following declining Gfi1b and LSD1 expression drives megakaryocytic differentiation. In contrast, in erythrocytes, robust Gfi1b and LSD1 expression [20] keeps the expression of these and perhaps other factors in check and ensures lineage fidelity by suppressing megakaryocytic gene expression.

## Materials and Methods

### Chromatin immunoprecipitation (ChIP)

ChIP experiments were performed in uninduced and induced L8057 cells as previously described [15, 19] with anti-Gfi1b (SCBT; # 8559) and anti-LSD1 (Abcam; #17721) antibodies. Primers used for qPCR amplification of ChIP DNA are listed in the Supplement.

### Plasmid construction and expression

Murine Talin1 (Accession Nos. NM_011602.5), Talin1 head domain (THD) and Kindlin3 cDNAs (Accession Nos. NM_153795.2) were PCR amplified from total RNA from L8057 cells and sub-cloned into the pCDH-MSCV^™^ lenti-viral expression vector (Systems Biosciences). Protein expression was confirmed in 293T cells. Commercially available Kindlin3 and Talin1 shRNAs were purchased from the Mission^™^ collection (Sigma Aldrich) and are listed in the Supplement. Promoter constructs were produced by PCR amplification of genomic DNA from wild type mouse tails followed by ligation into the luciferase reporter plasmid pGL3 (Promega).

### Cell culture and cell line production

L8057 (megakaryoblastic cell line) [29] was maintained as previously indicated [20] and induced to differentiate with 50nM 12-O-tetradecanoyl phorbol-13-acetate (TPA) for 5 days. K562 cells (ATCC^®^ CCL243^™^) [30] were cultured in IMDM media supplemented with 10% fetal bovine serum and antibiotics and also induced to differentiate into megakaryocytes with 50 nM TPA [31, 32]. Cells were harvested as needed for protein and RNA collection or histological staining.

### Transfection, luciferase and β-gal assays

Transfections were performed in HEK-293T cells, which were co-transfected with 1 μg of luciferase reporter, specified amounts of expression vectors and 50 ng of pEF4-β-gal. After 48 hours cells were harvested and luciferase activity determined using an assay reagent (E1500; Promega) on a Glorunner^™^ microplate luminometer (Turner Biosystems).–β-gal assay (E2000; Promega) was performed to normalize for transfection efficiencies.

### Culture and manipulation of fetal liver cells

Total fetal liver cells (~10^5^) from day 12.5 embryos (e12.5) were harvested and cultured directly or transduced with lenti-viruses encoding the indicated shRNAs or cDNAs. Cells were differentiated along the megakaryocytic lineage with thrombopoietin (20 ng/ml) and IL3 (10 ng/ml) and selected for vector retention with puromycin (0.5–1 μg/ml).

### Animal husbandry and manipulation

All mice were maintained, manipulated and euthanized in the CCNY vivarium as per the PI’s approved IACUC (Institutional Animal Care and Use Committee) protocol (# 982; expiration date: 9/15/18). 6–8 week old wild type (C57Bl/6) or mutant (*gfi1b-/-*; 129SV backcrossed into C57Bl/6) mice were subjected to timed matings to obtain staged embryos for tissue (fetal liver) collection. Adult mice were euthanized by asphyxiation with CO_2_ and palpitated to confirm death prior to dissections.

### Preparation of cell lysates and Western blotting

Cells were lysed in whole cell lysis buffer (50 mM Tris-HCl pH7.4, 150 mM NaCl, 1 mM EDTA, 1% Triton X-100). Lysates were resolved on SDS-PAGE and Western blotted with antibodies for Kindlin3 (Abcam; #ab68040), Talin1 (Abcam; #ab71333) and integrinβ3/CD61 (Abcam; #ab119992).

### qPCR, histological assays and flow cytometry

RNA expression was quantified by qPCR on an ABI 7500 machine (Applied Biosciences). Marker expression was normalized to HPRT (hypoxanthine phosphoribosyl transferase). qPCR primer sequences have either been reported previously [15] or are listed in the Supplement.

Histochemical analysis (benzidine, and acetylcholine esterase staining) was performed on ~10^5^ cells as previously described [1]. The number of positively staining cells relative to total for a fixed area were determined using the ImageJ^™^ cell imaging and counting software [33]. For flow cytometric analyses of surface markers, ≥10^5^ cells were stained with FITC-conjugated anti-mouse CD9 and APC-conjugated anti-mouse CD41 antibodies (eBioscience), respectively and analysis was performed on the BD LSRII Analyzer (Becton Dickinson). For FACS sorting, cells were labeled with lin–FITC and c-Kit–phycoerythrin (PE) and the lin^−^c-Kit^+^ population collected following elimination of dead cells, doublets and aggregates on a BD FACS Aria sorter (Becton Dickinson).

### Statistical analyses

The data for all qPCR reactions (from ChIP and RT) represents the mean±s.d. from three independent experiments. P values were calculated by one-way analysis of variance (ANOVA) (for comparison of three or more datasets) or by multiple **t**-test (for two datasets) followed by Holm-Sidak post-hoc test as applicable, for comparisons. Comparisons of parallel data series are designated by different symbols (*, §, +).

## Results

### Identification and characterization of *kindlin3* and *talin1* as Gfi1b transcriptional targets

**Fig 1:**
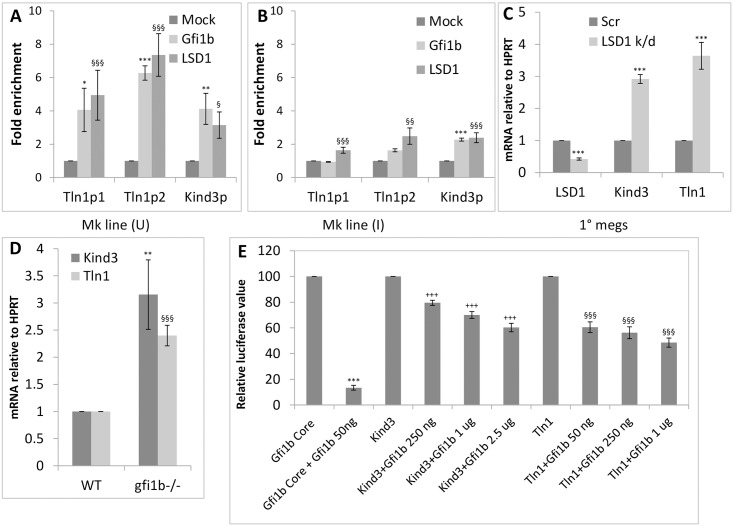
Regulation of Talin1 and Kindlin3 expression by Gfi1b and LSD1. A-B. Enrichment of Gfi1b and its co-factor LSD1 at two sites on the talin1 (Tln1p1 and Tln1p2) and one site (kind3p) on the kindlin3 promoter in undifferentiated (U; uninduced) (A) versus differentiated (I; induced) (B) L8057 cells (Mk line). C. Upregulation of Kindlin3 and Talin1 message in LSD1 inhibited primary megakaryocytes derived from in vitro culture of fetal liver cells (1° megs). D. Upregulation of Kindlin3 and Talin1 message in gfi1b-/- total fetal liver cells relative to wild type controls. E. Dose dependent repression of the isolated kindlin3 and talin1 promoters (sequence depicted in S1A and S1B Fig) by the indicated amounts of Gfi1b in HEK-293T cells. All graphs depict averages (solid bars) and standard deviations (error bars) from three independent experiments. P values are represented by various (*, §, +) symbols for different data series with values of < 0.05, 0.01 and 0.001 being indicated by one, two and three symbols, respectively.

The *talin1* and *kindlin3* promoters were identified as chromatin targets of Gfi1b and its cofactors LSD1 and CoREST/Rcor1 in previous ChIP-on-chip experiments in erythroid MEL (murine erythroleukemia) cells [15, 19]. The sequences of the promoter regions derived from the ChIP-on-chip experiments are shown in S1A and S1B Fig. Subsequent ChIP-qPCR analysis of putative Gfi1b binding sites in the promoters confirmed substantial enrichment of Gfi1b and LSD1 at these sites in the murine megakaryoblastic cell line L8057 [29] (Fig 1A), consistent with the robust expression of these factors at this stage of development (S2A Fig) [20]. Likewise enrichment of these repressor proteins on *talin1* and *kindlin3* promoters declined sharply upon phorbol ester mediated differentiation of L8057 cells into megakaryocytes (Fig 1B) commensurate with their reduced expression upon differentiation (S2A Fig).

Since Gfi1b and LSD1 are well known transcriptional repressors [6, 14, 15], the relative expression of Kindlin3 and Talin1 message in LSD1 inhibited and *gfi1b* mutant hematopoietic cells was interrogated relative to controls. As expected, both messages were strongly upregulated in LSD1 inhibited primary megakaryocytes derived from *in-vitro* culture of total fetal liver cells (Fig 1C) and in embryonic day 12.5 (e12.5) *gfi1b-/-* total fetal liver cells (Fig 1D) (representing a predominantly erythroid population [20, 34]), relative to their wild type counterparts.

To confirm direct repression of the *talin1* and *kindlin3* promoters by Gfi1b, promoter driven luciferase assays were performed in HEK-293T non-hematopoietic cells. Since the *gfi1b* promoter itself is most stringently repressed by its own protein product [35], a ~ 500 bp segment of the murine *gfi1b* promoter just upstream of the transcriptional start site and containing two closely spaced high affinity Gfi1b binding sites was used as a positive control in these experiments [35][19]. These assays demonstrated dose dependent and differential repression of the *kindlin3* and *talin1* promoters by Gfi1b (Fig 1E). Although both promoters were considerably less sensitive to Gfi1b mediated repression relative to the *gfi1b* core promoter itself, which the *talin1* promoter exhibited marginally greater sensitivity to Gfi1b repression relative to *kindlin3*. The difference in the sensitivities of the two promoters to Gfi1b repression may reflect the difference in the number (two in the *talin1* promoter versus one in the *kindlin3* promoter) and affinity (both the sites in the *talin1* promoter show greater enrichment for Gfi1b and LSD1 relative to the *kindlin3* site) (Fig 1A) of the Gfi1b/LSD1 binding sites in the two elements. These observations may further represent real differences in the responsiveness of the endogenous promoters to Gfi1b doses in erythro-megakaryocytic cells enabling differential Talin1 and Kindlin3 transcription in identical nuclear milieus. The relatively greater upregulation of Talin1 message relative to Kindlin3 in LSD1 inhibited cells provides additional support for this notion. Overall, these observations identify Kindlin3 and Talin1 as new Gfi1b targets whose transcription is repressed by this factor and the histone demethylase LSD1 in erythro-megakaryocytic cells.

**Fig 2:**
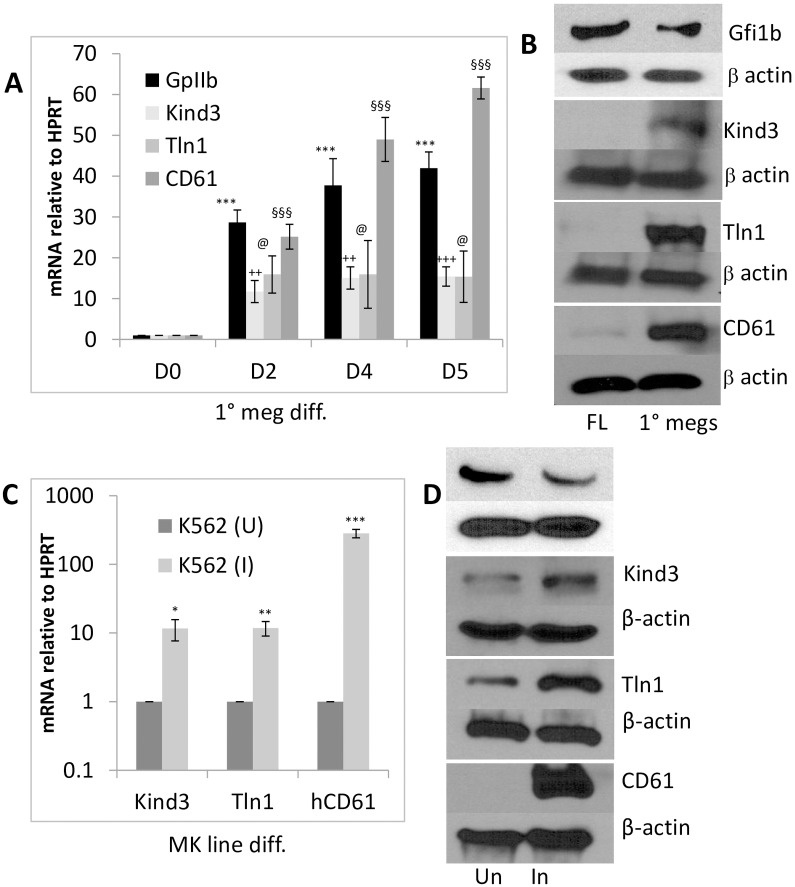
Up-regulation of Talin1, Kindlin3 and integrin expression upon megakaryocyte differentiation. A. Time course of Talin1, Kindlin3 and integrins GPIIb/CD41 and CD61 expression during in vitro megakaryocytic differentiation of total fetal liver cells. B. Gfi1b, Kindlin3, Talin1 and CD61 protein levels relative to β-actin (loading control) in freshly isolated total fetal liver cells (FL) versus megakaryocytes derived from them following in vitro culture (1° megs). 20–30 μg of total protein was loaded per lane. C. Up-regulation of kindlin3, talin1 and CD61 expression in the human hematopoietic cell line K562 uninduced (U) or induced to differentiate into megakaryocytes (I) with phorbol ester. D. Western blot of Gfi1b and corresponding proteins relative to β-actin. 60 μg of total protein was loaded per lane. All graphs depict averages (solid bars) and standard deviations (error bars) from three independent experiments. Western results of one from three representative experiments are shown. P values are represented by various (*, §, +) symbols for different data series with values of < 0.05, 0.01 and 0.001 being indicated by one, two and three symbols, respectively.

Accordingly, consistent with the previously documented and recently revalidated decline in Gfi1b expression during the maturation of primary megakaryocytes and cell lines [20] (Fig 2B and 2D; top panels and S2B Fig), both Gfi1b gene targets as well as integrinβ3/CD61 showed a steady and reciprocal increase in both message and protein expression in these cells (Fig 2A and 2B). A similar increase in Kindlin3, Talin1 and Integrinβ3/CD61 message and protein expression were also observed upon phorbol ester driven megakaryocytic differentiation [36] of the human multi-potent hematopoietic cell line, K562 (Fig 2C and 2D) and the murine megakaryoblastic cell line L8057 (S3A Fig). These results confirm repression of Kindlin3 and Talin1 transcription by Gfi1b and the reciprocal expression of these cytoskeletal proteins relative to their repressor during megakaryocytic differentiation.

### Stimulation of megakaryocytic differentiation by Kindlin3, Talin1 and Integrinβ3

**Fig 3:**
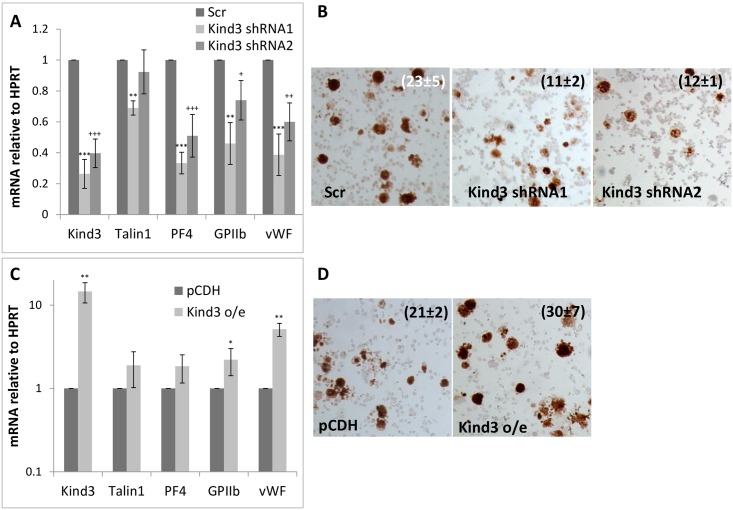
Effect of manipulating Kindlin3 expression on megakaryocytic differentiation. A. Kindlin3, Talin1 and megakaryocytic marker message levels in control (Scr; scrambled) and Kindlin3 knocked down fetal liver cells differentiated into megakaryocytes. B. Acetyl choline esterase staining of the corresponding cells. P values ranged from <0.01 to <0.001 for positive cell counts. C. Message levels of indicated factors in control (pCDH; empty vector) and Kindlin3 over-expressing fetal liver cells differentiated into megakaryocytes. P values are represented by various (*, §, +) symbols for different data series with values of <0.05, 0.01 and 0.001 being indicated by one, two and three symbols, respectively. D. Acetyl choline esterase staining of the corresponding cells. The mean ± s.d. of acetylcholine-positive cells as a percentage of the total population from three independent experiments is indicated. P value was determined to be <0.05.

**Fig 4:**
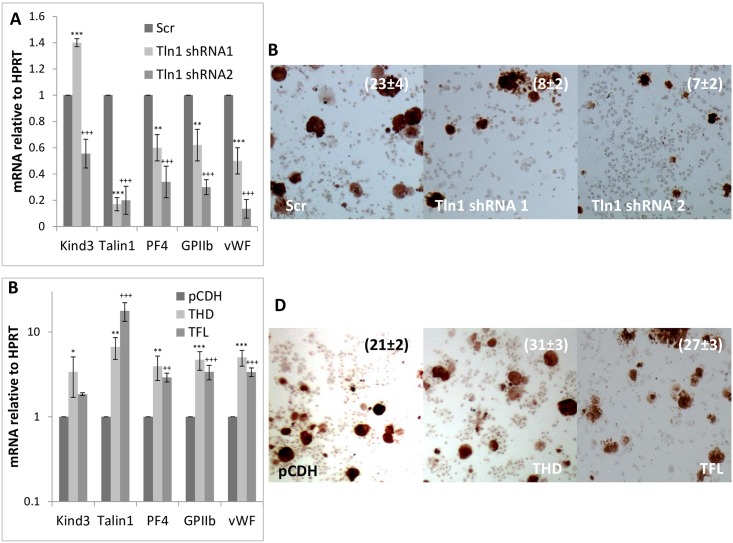
Effect of manipulating Talin1 expression on megakaryocytic differentiation. A. Kindlin3, Talin1 and megakaryocytic marker (platelet factor 4, glycoprotein IIb/CD41 and von Willebrand factor) message levels in control (Scr; scrambled) and Talin1 knocked down fetal liver cells differentiated into megakaryocytes. B. Acetyl choline esterase staining of the corresponding cells. P values were <0.001 for the cell counts. C. Message levels of indicated factors in control (pCDH; empty vector), Talin1 head domain (THD) and full length (TFL) over-expressing fetal liver cells differentiated into megakaryocytes. P values are represented by various (*, §, +) symbols for different data series with values of < 0.05, 0.01 and 0.001 being indicated by one, two and three symbols, respectively. D. Acetylcholine esterase staining of the corresponding cells. The mean ± s.d. of acetylcholine-positive cells as a percentage of the total population from three independent experiments is indicated. P values ranged from <0.05 to <0.01.

**Fig 5:**
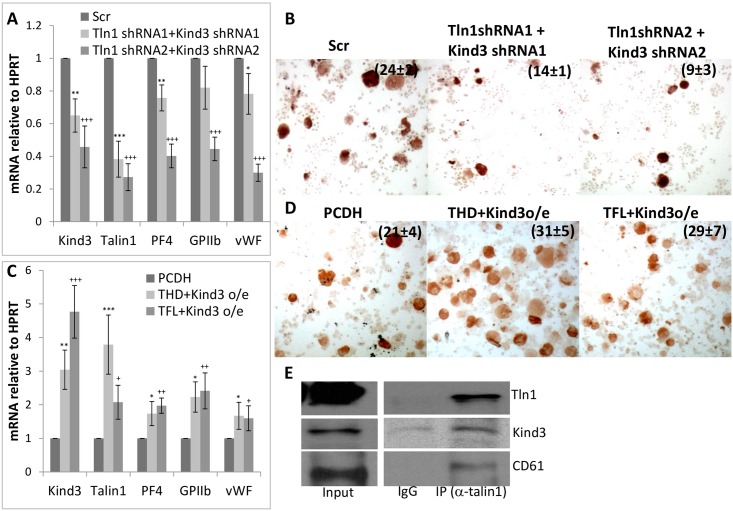
Effect of combined manipulation of Talin1 and Kindlin3 expression on megakaryocytic differentiation. A. Kindlin3, talin1 and megakaryocytic marker message levels in control (Scr; scrambled) versus Talin1 and Kindlin3 knocked down fetal liver cells differentiated into megakaryocytes. B. Acetyl choline esterase staining of the corresponding cells. P values were <0.001. C. Message levels of indicated factors in control (pCDH; empty vector), Talin1 head domain (THD) and Kindlin3 and Talin1 full length (TFL) and kindlin3 over-expressing fetal liver cells differentiated into megakaryocytes. P values are represented by various (*, §, +) symbols for different data series with values of < 0.05, 0.01 and 0.001 being indicated by one, two and three symbols, respectively. E. Immunoprecipitation of kindlin3 and CD61 with Talin1. Input represents 10% of lysate used for the immuno-precipitation. D. Acetyl choline esterase staining of the corresponding cells. The mean ± s.d. of acetylcholine-positive cells as a percentage of the total population from three independent experiments is indicated. P values were <0.05.

Although *kindlin3* and *talin1* are known to be required for platelet production and function, their individual deletions are not known to drastically impact megakaryopoiesis [22, 23]. However, to determine the effect of Kindlin3 and Talin1 protein levels in megakaryocytic differentiation, we inhibited or over-expressed their mRNAs in primary megakaryocytes. As depicted in Figs 3–5, inhibition of Kindlin3 and Talin1 either individually or in combination diminished megakaryocytic differentiation as assessed from differentiation marker (GPIIb, PF4 and vWF) expression and histochemical analysis (acetyl choline esterase activity) (Figs 3A, 3B, 4A, 4B, 5A and 5B) while over-expression produced the opposite results and enhanced differentiation (Figs 3C, 3D, 4C, 4D, 5C and 5D).

Interestingly, expression of the Talin1 head domain, known to be necessary and sufficient for integrin activation in platelets and other cells [28, 37] appeared to be relatively more potent at driving differentiation relative to the full length protein (Figs 4D and 5D). This result suggests that not only is the remainder of the Talin1 protein, namely the Talin1 rod domain, which binds actin and vinculin, dispensable for the function of Talin1 in promoting megakaryocytic differentiation, but that removing this domain moderately enhances the potency of the THD in mediating integrin activation and cellular differentiation. This observation in conjunction with the interaction between Kindlin3, Talin1 and integrinβ3 in megakaryocytes (Fig 5E) demonstrates physical co-operativity between these factors in producing integrin activation. The cytoskeletal maturation resulting from the increased expression and cooperative actions of these factors then constitutes a major driving force in megakaryocytic differentiation.

**Fig 6:**
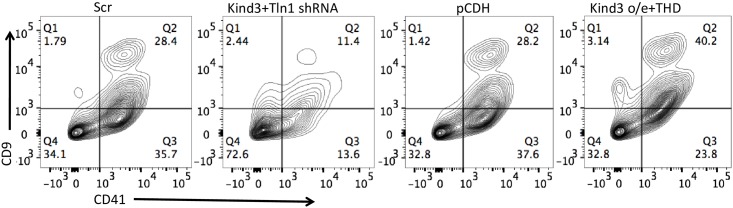
Effect of manipulating Talin1 and Kindlin3 expression in hematopoietic progenitors. Expression of the megakaryocytic cell surface markers CD9 and CD41 in c-kit+lin- hematopoietic progenitors isolated from e13.5 fetal liver cells and cultured in vitro following transduction with the indicated shRNAs and cDNAs. Results depict one of two independent experiments.

To confirm that the enhancement in megakaryocytic differentiation by Kindlin3 and Talin1 occurred at the level of megakaryocytic erythroid progenitors (MEPs) and not simply lineage committed cells, c-kit^+^lin^-^ hematopoietic progenitors isolated from embryonic day 12.5–13.5 fetal livers were transduced with talin1 and kindlin3 shRNA and cDNAs and following *in-vitro* culture with megakaryocytic cytokines analyzed for the expression of the surface markers CD9 and CD41 (Fig 6). These observations further confirmed stimulation of megakaryocytic differentiation and vice versa by Kindlin3 and Talin1, particularly the talin1 head domain.

The quantitative differences in megakaryocytic differentiation following manipulation of Kindlin3 and Talin1 as seen in our assays, contrast the relative lack of reported megakaryocytic phenotypes in mice lacking these factors. This may either be due to modest quantitative deficits in these processes that were not specifically detected in these animals or from compensatory mechanisms in the surviving animals that minimized phenotypic deficiencies in megakaryocytic development in the knockouts.

## Discussion

### Reciprocal expression of Gfi1b and its gene targets in regulating erythroid and megakaryocytic differentiation

Megakaryocytes and their derivative platelets are essential for survival and homeostasis. Yet the molecular programs and players involved in the emergence and differentiation of megakaryocytes remain partially defined. Here we identify the cytoskeletal proteins Kindlin3 and Talin1 as major gene targets of the Gfi1b transcriptional repressor complex. Reciprocal upregulation of these proteins following declining Gfi1b and LSD1 expression during megakaryocyte maturation, stimulates differentiation of these cells, likely by co-operatively activating integrins (whose expression also increases concomitantly) and priming these cells for proplatelet and platelet production.

Gfi1b was previously shown to be essential for the generation of both erythroid and megakaryocytic cells [1] [2]. However, our recent observations, now demonstrate that although this transcriptional repressor is essential for the specification of these lineages from a common progenitor, its expression declines steadily following the initiation of the megakaryocytic lineage i.e. the production of megakaryoblasts [20]. This in turn enables up-regulation of diverse megakaryocyte specific, lineage promoting, factors like Meis1 [19], Rgs18 and perhaps other Rgs factors [20] and cytoskeletal proteins like Kindlin3 and Talin1 (this study) that then collectively promote differentiation of these cells.

In contrast, robust and sustained expression of Gfi1b (and its co-factor LSD1) well into the erythroid differentiation program (S2A Fig) [20] may be needed to maintain lineage fidelity by suppressing megakaryocytic gene expression in this closely related alternative lineage. However, whether Gfi1b is altogether dispensable for megakaryocytic differentiation beyond the megakaryoblast stage, or if a low but finite level of this factor is required for megakaryocyte differentiation awaits the lineage and stage specific deletion of *gfi1b* in mice carrying a floxed allele of this gene [2].

### Co-operativtivity between cytoskeletal and signaling processes in megakaryocytic differentiation

Curiously, our Talin1 over-expression experiments demonstrate that the Talin1 head domain is relatively more potent than the full-length protein in promoting megakaryocytic differentiation. Since the THD is known to activate integrin β3 by tilting its transmembrane domain (TMD) [28, 37], our observations demonstrate that this process is sufficient for stimulating megakaryocyte differentiation. These results further indicate that Talin1 interaction with other cytoskeletal proteins such as actin and vinculin, which are mediated by the rod domain, are dispensable for Talin1 mediated stimulation of differentiation. Moreover the greater potency of the THD in stimulating differentiation suggests either a greater affinity of the truncated protein for integrinβ3 and/or an increased ability of the THD to tilt the integrinβ3 transmembrane domain and activate it. Either of these effects could be due to the lack of steric constraints on the THD relative to the full-length protein due to the latter being tethered to actin or other cytoskeletal proteins.

Both in platelets and in megakaryocytes, increased expression of integrins coupled with their heightened “inside out” activation upon association with abundant Kindlin3 and Talin1 then enables high affinity multi-meric ligand binding. Ligand engagement in turn produces “outside-in” integrin signaling which activates downstream cascades like the PI3K (phosphatidyl inositol 3-OH kinase)/Akt pathway [38]. In megakaryocytes, activated Akt phosphorylates FOXO proteins leading to their nuclear exclusion, which alleviates their repression of the Notch pathway transcription factor, RBP-Jκ, and promotes megakaryocytic differentiation [39, 40] (S3B Fig).

Curiously, our recent screen for Rgs18 (another robust gene target of Gfi1b which potently stimulates megakaryocyte differentiation) interacting proteins has uncovered extensive associations of this GAP (GTPase activating protein) with Kindlin3, Talin1, Rap1 (Ras-proximate-1) and multiple integrins and integrin associated proteins in megakaryocytes (Sengupta and Saleque; unpublished observations). Since Rap1, a Ras-like small GTPase and its associated protein RIAM (Rap1-GTP interacting adaptor molecule) are important for recruiting Talin1 to integrinβ tails [41, 42], these preliminary observations suggest the existence of an elaborate cytoskeletal associated signaling network which may synergistically drive megakaryocytic differentiation by funneling multiple and diverse extracellular signals to promote this process. The exact nature, sequence and consequences of these interactions on megakaryocytic development and function currently awaits a more systematic investigation of these emergent processes, followed by determining the consequences of disrupting of one or more of the most significant interactions and/or protein mediators, identified by these analyses.

### Developmental and clinical relevance

These studies could produce a wealth of information on both megakaryocyte differentiation and platelet aggregation thus providing not only a more complete picture of these vital processes but also revealing more avenues for regulating them in disorders caused by their hypo- or hyper-activity or dysfunction. Moreover, since Kindlins, Talins and Integrins are expressed in a wide spectrum of cells, insights into their co-operativity and coordinated functions should be relevant in other developmental contexts and in diseases associated with the dysfunctions of these proteins or their paralogs in these tissues. Remarkably Talin1 is known to be significantly up-regulated in metastatic cells of several cancers such as prostate cancer, breast cancer, hepatocellular carcinoma etc. [43–45]. Therefore a fuller understanding of the molecular mechanisms underlying Talin1 mediated cellular adhesion and motility will reveal possible avenues for disrupting its downstream effects like blocking its binding to, and activation of integrins, and/or by inhibiting Akt signaling.

## Supporting Information

S1 Fig Annotated sequences of the *talin1* and *kindlin3* promoter sequences obtained by ChIP-on-chip. **A.** Murine *talin1* promoter sequence (Accession # JN945242). Sequence included in promoter construct driving luciferase expression is indicated in black. Exon1 sequence is shown in bold letters with the transcription start site (tss) underlined. Primers used for ChIP qPCR are underlined and the putative Gfi1b binding sites shaded. Tln1p1 represents the 5’ and Tln1p2 the 3’ amplicons in Fig 1A and 1B, resepctively. **B.** Murine *kindlin3* promoter sequence (Accession # JN958519). Sequence included in promoter construct is indicated in black. The initiator ATG is underlined. Primers used for ChIP qPCR are underlined and the putative Gfi1b binding site is shaded. The sequence is numbered relative to the tss (not shown).
(PDF)

S2 Fig Gfi1b protein and message expression upon erythro-megakaryocytic differentiation. **A.** Gfi1b, LSD1 and β-actin protein levels in immature (U) and mature (I) megakaryocytes (L8057; Mk line) (left panel) and erythroid (murine erythroleukemia [MEL]; ery line) cells (right panel). **B.** Time course of GPIIb, Gfi1b and Rgs18 (another Gfi1b target) message expression in fetal liver cells differentiated in culture into megakaryocytes (1° meg diff). These figures were reproduced from [20].
(PDF)

S3 Fig Regulation of megakaryocytic differentiation by Gfi1b and its cytoskeletal effectors. **A.** Kindlin3, Talin1 and CD61 protein expression in megakaryocytes. Western blot of Kindlin3, Talin1 and CD61 expression in uninduced (Un) and induced (In) L8057 cells. 60 μg of total protein was loaded per lane. **B.** Model of regulation of megakaryocytic differentiation by Gfi1b, Kindlin3, Talin1 and their downstream effectors.
(PDF)

S1 File Supporting Materials and Methods. (PDF)
