# Supplementary material for: Cooperative Stimulation of Megakaryocytic Differentiation by Gfi1b Gene Targets Kindlin3 and Talin1
Source: PLoS One. 2016 Oct 21;11(10):e0164506. doi: 10.1371/journal.pone.0164506 (PMC5074496; doi:10.1371/journal.pone.0164506)
Supplement: S1 Fig — A. Murine talin1 promoter sequence (Accession # JN945242). Sequence included in promoter construct driving luciferase expression is indicated in black. Exon1 sequence is shown in bold letters with the transcription start site (tss) underlined. Primers used for ChIP qPCR are underlined and the putative Gfi1b binding sites shaded. Tln1p1 represents the 5’ and Tln1p2 the 3’ amplicons in Fig 1A and 1B, resepctively. B. Murine kindlin3 promoter sequence (Accession # JN958519). Sequence included in promoter construct is indicated in black. The initiator ATG is underlined. Primers used for ChIP qPCR are underlined and the putative Gfi1b binding site is shaded. The sequence is numbered relative to the tss (not shown). (PDF) [file pone.0164506.s001.pdf]

S1 Fig:

A

CACAACTTATCATTTCACACTCACCTAGATCTATGGAGACGTGCTCCTGTGGAAGGAGT −587

AGTTGTGATCATGGAGAATGGAAGAGCTGGAAGAGCCGAGAACATCCCGCGATACTGAGG −527

GACTGAGCAGAGAGCTCAGTAAATCCTCTACCTCCCAGTCGCTCAGTTCTTGCACGGAGT −467

TCTATCAAATTCAAAGGCCTTGATTTATTTCCGTACCTTGGGTCTCGCGGACATCCCTAG −407

CTTCCCCCACTGGGAACACCCACCCACCCACCTCGGAAGGTGACAGCTCTAAGTCCAGCG −347

GAGCCTTCACGTCCGGCTCAGTCGCAGCCCACAAATCTCCGCTTTCCTCTAGCAGGAAGC −287

CCAGCAGGTCCTGATCACCGGGATCCAAAGCTAGCAGATCCTGACCACCAGGATCCATTT −227

GGGATATCTATGGATAGAAATCCCTCCGCGCAGATCCAACCTCCGAACCTCAGGCACGAC −167

CCCTCAGTCTGTCCAGTCGGCTCGACTTAACCTCCGTCACTCCGGGACAAGGGCCTAGCT −107

GTCACTCTAAACCGCACCCGTCTCTCAGCTTCTGTATTCCTCAGCTTCCAACACAACGGA −47

AGTGGTGCACATAGCCCCGTGGAATCCTGGGACTTGGAGTTCTCTCC**AACTCGGAAGTGG** **+13**

CTCCTGGGCTGCGCCACGTCCCGGGGGCTATGCAAATTATGGGGACGTCCTTTCAAGCTT **+73**

CCCACGCCCCAGGGCGGGGATTCTGAGGATCCTTGTCCGCCTTCGCCTCGAGCTATTAAA +133

AAAAAAAACTTAAGCGTTTAAAAGGGAACTCCAGCTTAGTTCGGGCGTTCTCTCCAGAGC +193

TCTGCACGCTCGCTTCCTGCTGGGGAGGGGCGGCCAGGCTTCGCGGGCGCCCGAGCATCG +253

AGAACTACGGCCAGAGCAGCTTCCTGCAGTCTCCGCTACCATAGAGCGCGGGCCCAGGGC +313

GCCGCCGGCGGGTGGGGGACGTTTCCAGGACGGAAGTGGCCGAGAGTGTGTCGAAGGGAG +373

GGCGAGGCCGGAGCCCCAAAAGCGACCGGGAGAAGGAGCGGGTAGCGGGCCCAGGGCGGG +433

GCGCAGAGCCGGGCAGCGCAGGTAGGCCGGACGGGCTGGGCCGAGCTTCTAGGTGGGGCT +493

B

CCGCAGCC**ATG**GCGGGTATGAAGACAGCCTCCGGGGACTATATCGACTCTTCCTGGGAGC +572

TGAGGGTGTTTGTGGGCGAGGAGGACCCTGAGGCCCAGTCTGTCACACTCCGAGTCACGG +632

GGGAGTCGCACATTGGTGGGGTACTTCTGAAGATCGTGGAGGAAATCAGTGAGTCCCCGT +692

GACCCTATGTATCCAGTCATGACAGCTCTCCCTGGCTTTTCTAGGCCATTCTCGCCTCTA +752

CTCATAACCTTGTCACAGTGACTCAGGCAACAGTCTTTAATTATTTGGTTCCAAAGAAGG +812

TATTTCTTCAACTTCCCCTCTCCATACTTCCTCCAGCTCCTACTGGTTCTGTCTAGTCTC +872

CAAGAGACAGACAACATGGTCCCCTGTGTGTCTTGCCTGTGCCGAGGCTTATTATGTTCC +932

TGAGGGATGGCTAGGAGGTTCCTGGGGACCTGGCACCTACTCTCCTAAATTTCCTCCCCA +992

GCAAACCACTTGGCAGGCTGCTGACAGTGGGTGAGAGGCCCTGCCCCCACAGGAAGTTAC +1052

GGCAGAGTAGGCTAGGGCGATATCAGTCACCCAGAAGGCCCCATGAGGCTTCTCATCAAC +1112

TTTACCAGCAGAGAATGAGAGTTATCACTTCCTGGGAGGATGGCAGGAAAGCCCCGATAG +1172

TCAGAGCCGGAAGACATCCCACCATGAGCTCCCGATAGTCAGAGCCGGAAGACATCCCAC +1232

CATGAGCTCCCGATAGCCAGAGACAGAAGGCATCTCACCATGAGTGTGGGTCCTAGGCCT +1292

CTGGAATTGGGACAGGGATAGAAGGCAGGTTTTCTAGACCCACCCAACCCTCTATGTGTA +1352

TGCAGTCCAAGACGGACCAGAGTGACTGAGCAGGACAAGCCCCAACACAGCCAAATAACA +1412

GGGTTGAGACAGAAGGGAACTGCCGGAAGCAGGGCCCAAAGAGGCTGTGAAAGCAGAAGT +1462

GGGAAGAGGGTTAGTCCCTCAGAAACTGGA +1492
