# Supplementary figures and images for: Cooperative Stimulation of Megakaryocytic Differentiation by Gfi1b Gene Targets Kindlin3 and Talin1
Source: PLoS One. 2016 Oct 21;11(10):e0164506. doi: 10.1371/journal.pone.0164506 (PMC5074496; doi:10.1371/journal.pone.0164506)

S2 Fig:

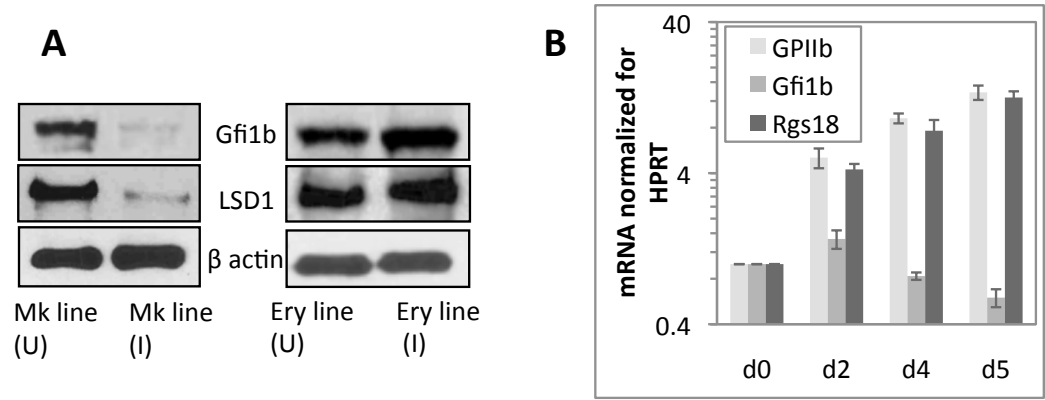

Supplement: S2 Fig — A. Gfi1b, LSD1 and β-actin protein levels in immature (U) and mature (I) megakaryocytes (L8057; Mk line) (left panel) and erythroid (murine erythroleukemia [MEL]; ery line) cells (right panel). B. Time course of GPIIb, Gfi1b and Rgs18 (another Gfi1b target) message expression in fetal liver cells differentiated in culture into megakaryocytes (1° meg diff). These figures were reproduced from [20]. (PDF) [file pone.0164506.s002.pdf]

**S3 Fig:**

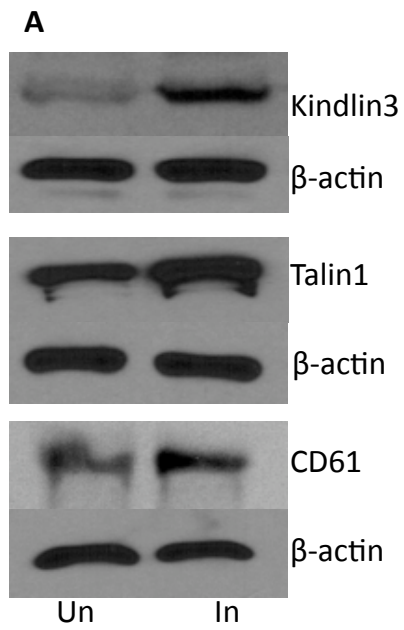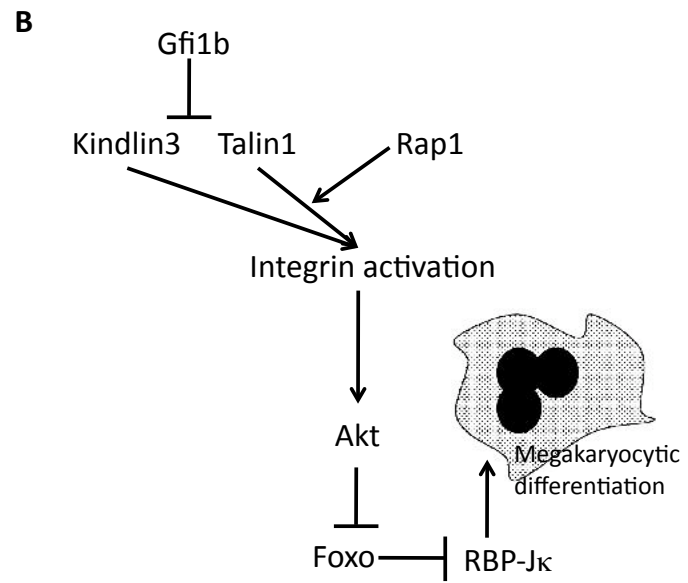

Supplement: S3 Fig — A. Kindlin3, Talin1 and CD61 protein expression in megakaryocytes. Western blot of Kindlin3, Talin1 and CD61 expression in uninduced (Un) and induced (In) L8057 cells. 60 μg of total protein was loaded per lane. B. Model of regulation of megakaryocytic differentiation by Gfi1b, Kindlin3, Talin1 and their downstream effectors. (PDF) [file pone.0164506.s003.pdf]
