# Supplementary material for: Cooperative Stimulation of Megakaryocytic Differentiation by Gfi1b Gene Targets Kindlin3 and Talin1
Source: PLoS One. 2016 Oct 21;11(10):e0164506. doi: 10.1371/journal.pone.0164506 (PMC5074496; doi:10.1371/journal.pone.0164506)
Supplement: S1 File — (PDF) [file pone.0164506.s004.pdf]

Supporting Materials and Methods:

ChIP-qPCR primers:

Talin promoter site 1 (5’): CACAACTTATCATTTCACACTCACC and GAGCTCAGTAAATCCTCTA

Talin promoter site 2 (3’): ACAAATCTCCGCTTTCCTCTAGC and TCCATTTGG GATATCTATG

Kindlin3 promoter: GGGGACTATATCGACTCTTCCTG and ACTCACTGATTTCCTCCACGAT and

S_{μ}: CTTGAGCCAAAATGAAGTAGACTGT and ACAGTCCAGTGTAGGCAGTAGAGTT.

Primers for expression vector construction:

Talin1 (full length): ATGGTTGCGCTTTCGCTGAAGATT and GTGCTCGTCTCGAAGCTCTGAAG

Talin1 Head: ATGGTTGCGCTTTCGCTGAAGATT and CTGAAGGACTGTTGACTTTTT

Kindlin 3: ATGGCGGGTATGAAGACAGCCTCC and GAAGGCCTCATGGCCTCCTGTAAG

shRNA sequences:

Kindlin3 shRNA1: CCGGGAGGAACCACAAATCCTGGTTCTCGAGAACCAGGATTTGTGGTTCCTCTTTTTTG

Kindlin3 shRNA2: CCGGGCTGGATAGTCTCACTACCATCTCGAGATGGTAGTGAGACTATCCAGCTTTTTTG

Talin1 shRNA1: CCGGGCCCATTGTAATCTCTGCTAACTCGAGTTAGCAGAGATTACAATGGGCTTTTTG

Talin1 shRNA2: CCGGCGCTCCAAGAGTATTATTAATCTCGAGATTAATAATACTCTTGGAGCGTTTTTG

Primers for luciferase reporter construction:

Talin1 promoter:

AATACGCGTACCTTGGGTCTCGCGGACATC and ATTCTCGAGGAAGGCGGAGAAGGATCCTCAGAA

Kindlin3 promoter : GGGTATGAAGACAGCCTCCGGG and TCCAGTTTCTGAGGGACTAACCCT

qRT-PCR primers:

Mouse Talin1: GAGAGCTGATGGAAGAAAAGAAAGA and AGCTTCTCCATCTTCTTTTCA TCTC

Mouse integrin b3: GCCATTATGTTTACAGAGGACATTT and CGTCACACACATAAGTACACACAGA

Human Kindlin3: ATC AAT CGCA AGC AGG ACT GGT and TGA GGA GGC GGC AGA TGG CAG CC

Human Talin1: GCTGAGCCCCGTCAGAACCTGC and ACACTCTGGCCTTGAGGACCAG
